# Different directional energy dissipation of heterogeneous polymers in bimodal atomic force microscopy

**DOI:** 10.1039/c9ra03995c

**Published:** 2019-09-02

**Authors:** Xinfeng Tan, Dan Guo, Jianbin Luo

**Affiliations:** State Key Laboratory of Tribology, Tsinghua University Beijing 100084 China guodan26@tsinghua.edu.cn luojb@tsinghua.edu.cn

## Abstract

Dynamic force microscopy (DFM) has become a multifunctional and powerful technique for the study of the micro–nanoscale imaging and force detection, especially in the compositional and nanomechanical properties of polymers. The energy dissipation between the tip and sample is a hot topic in current materials science research. The out-of-plane interaction can be measured by the most commonly used tapping mode DFM, which exploits the flexural eigenmodes of the cantilever and a sharp tip vibrating perpendicular to the sample surface. However, the in-plane interaction cannot be detected by the tapping mode. Here a bimodal approach, where the first order flexural and torsional eigenmodes of the cantilever are simultaneously excited, was developed to detect the out-of-plane and in-plane dissipation between the tip and the polymer blend of polystyrene (PS) and low-density polyethylene (LDPE). The vibration amplitudes and phases have been recorded to obtain the contrast, energy dissipation and virial *versus* the setpoint ratio of the first order vibration amplitude. The pull-in phenomenon caused by a strong attractive force can occur near the transitional setpoint ratio value, the amplitude setpoint at which the mean force changes from overall attractive to overall repulsive. The in-plane dissipation is much lower than out-of-plane dissipation, but the torsional amplitude image contrast is higher when the tip vibrates near the sample surface. The average tip-sample distance can be controlled by the setpoint ratio to study the in-plane dissipation. Both flexural and torsional phase contrasts and torsional amplitude contrast can also be significantly enhanced in the intermediate setpoint ratio range, in which compliant heterogeneous materials can be distinguished. The experiment results are of great importance to optimize the operating parameters of image contrast and reveal the mechanism of friction dissipation from the perspective of in- and out-of-plane energy dissipation at different height levels, which adds valuable ideas for the future applications, such as compliant materials detection, energy dissipation and the lateral micro-friction measurement and so on.

## Abstract 2

Dynamic force microscopy (DFM) has become a multifunctional and powerful technique for the study of the micro–nanoscale imaging and force detection, especially in the compositional and nanomechanical properties of polymers.

## Graphical abstract

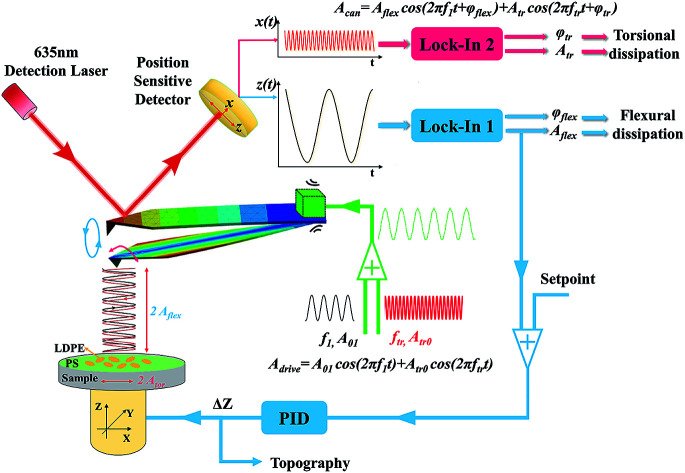

## 1. Introduction

In recent years, dynamic force microscopy (DFM) has become a multifunctional and universal technique for the application of micro–nanoscale imaging and force detection, including topography imaging, and measuring modulus of elasticity, viscoelastic properties and other physical properties in the microscale and nanoscale worlds.^1–7^ The new techniques in the DFM field, such as bimodal^8–11^ or higher modes,^12–14^ multi-frequency AFM,^15–17^ and intermodulation method,^18–20^ have complemented the traditional amplitude modulation mode and can obtain high resolution images of heterogeneous materials, cells or DNA.^21^ In bimodal mode, the cantilever is often excited by the first and second flexural resonance frequencies simultaneously. The mixed response signals of the cantilever are processed in two lock-in amplifiers to obtain the peak values. The first order flexural vibration amplitude is utilized for the topographical feedback and the corresponding phase is used for the out-of-plane dissipation. Meanwhile, the amplitude and phase of the second order flexural vibration are also exploited to detect the changes in mechanical, magnetic or electrical properties of the sample surface.^22–24^

The general form of bimodal AFM is the first two excitation flexural frequencies, indicating that the out-of-plane interaction can be explored by this method. However, sometimes the friction, the in-plane interaction or dissipation play an important role in the detection of the heterogeneous materials and some laminated structures,^25,26^ so it is necessary to excite the torsional vibration mode to detect the local mechanical and tribological properties in lateral dimension.^27,28^ In previous researches, the bimodal method was often implemented in ultra-high vacuum (UHV) or liquid for the high resolution. The ultrasensitive detection of lateral atomic-scale interactions on graphite (0001) has been completed *via* room-temperature dynamic force microscopy using simultaneous excitation and FM detection of the lowest flexural and torsional cantilever resonance modes.^26^ In addition, the frictional processes on the Br-doped NaCl (001) surface in the torsional channel revealed how the energy dissipates by the rearrangement of the tip apex and how the process is ultimately governed by lateral forces.^29^ For high resolution measurements, surface-normal and surface-parallel force components above the Ge(001) dimer surface and their direction-dependent anisotropy have been also expressed as a three-dimensional force vector distribution.^30^ For DFM, some novel excitation methods, such as the photothermal excitation method, have excellent performance in the bimodal DAFM and enhance the image quality.^31–34^ Especially in liquid environments, the use of torsional modes by the photothermal excitation method provides additional surface information revealing specific surface features, such as oxygen surface atoms on the calcite (101̄4) plane, enhancing the chemical surface contrast at the atomic level.^33^

However, considering the limited samples and experimental conditions, the technique should also be studied thoroughly in general atmospheric environment, where the scanning amplitude cannot be too low to maintain the stable feedback experiments due to the presence of water film on the sample surface. The in-plane dissipation has not been studied widely in the bimodal DFM. For example, the interaction is controlled by the setpoint ratio in the vertical direction, however, when at different setpoint levels, the in-plane dissipation is likely to be different. The mechanism of the dissipation should be revealed in the experiments and analysis. On the other hand, the image discrimination is also an essential aspect for the heterogeneous materials. The measurement capability of the bimodal DFM can be tested in the amplitude and phase contrast comparison.

In this article, a bimodal approach, where the first order flexural and torsional eigenmodes of the cantilever are simultaneously excited, was developed to detect the contrast, the out-of-plane and in-plane dissipation (flexural and torsional dissipation) between the tip and the polymer blend of polystyrene (PS) and low-density polyethylene (LDPE). The flexural and torsional signal spectra were obtained by sweeping frequency and the resonance peaks were distinguished. Typically, the approaching tip first senses the attractive force, and then the mixture of the attractive force acting on the tip body and the repulsive force between the tip and sample surface. The amplitude and phase of the flexural and torsional response signals have been recorded and analyzed to calculate the contrast, energy dissipation power and virial at various setpoint ratios. The free flexural amplitudes have been varied to explore the effects of amplitudes on the above physical quantities. The pull-in phenomenon caused by a strong attractive force, such as van der Waals' force, the electrostatic force and the possible capillary force from a surface water film can occur near the transitional setpoint ratio value, which is the amplitude setpoint at which the mean force changes from overall attractive to overall repulsive, in the first order vibration amplitude because of the small force constant of the cantilever.^1^

## 2. Experiments

**Fig. 1:**
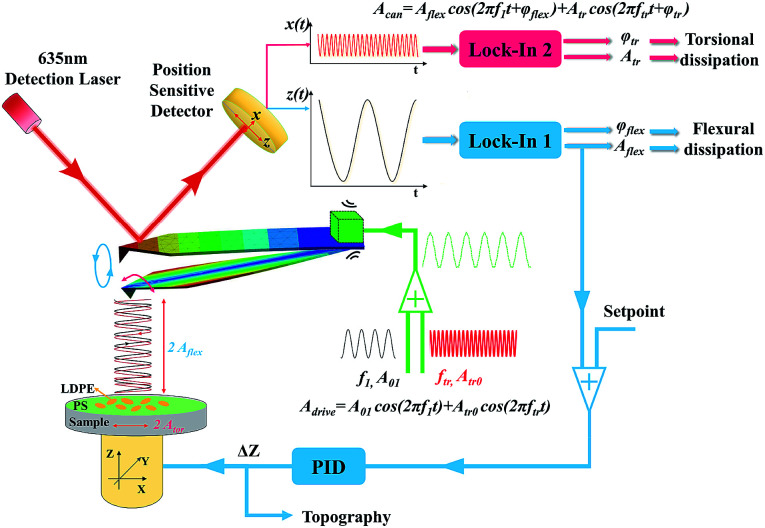
Bimodal schematic description and control system. The two excitation frequencies are the first order flexural and torsional mode frequencies, respectively. The response of the cantilever contains two frequency components same as the excitation frequencies. The flexural signal is used for topography feedback and the flexural dissipation, while the torsional signal is utilized to detect the torsional dissipation.

The thorough analysis has been conducted to reveal the attractive and repulsive interaction range and contrast transitional point of the polymer blend of polystyrene (PS) (*E*_PS_ = 2.0 GPa) and low-density polyethylene (LDPE) (*E*_LDPE_ = 0.1 GPa). The AFM used here is the NTEGRA, NT-MDT, Russia. The bimodal schematic description and control system are designed in Fig. 1, where the two dimensional photoelectric position-sensitive detector (PSD) is used to detect the vibration of the red laser (635 nm, 1 mW) reflected from the back of the cantilever, sensing the first order flexural and torsional mode frequency, amplitude and phase, respectively. The first order flexural and torsional mode are excited by the piezoelectric actuator, the reason of which is mainly the mechanical coupling of flexural and torsional modes.^35,36^ When simultaneously driving the piezo shaker at both the first order flexural and torsional frequencies, the cantilever responses are not usually only in the flexural mode, but also the torsional mode.^30^ The amplitude, frequency and phase of the mixed bimodal signals can be extracted by the two lock-in amplifiers to be calculated into contrast, energy dissipation and virial. The flexural vibration signal is used to control the piezo stage movement following the sample topography in the feedback loop.

**Fig. 2:**
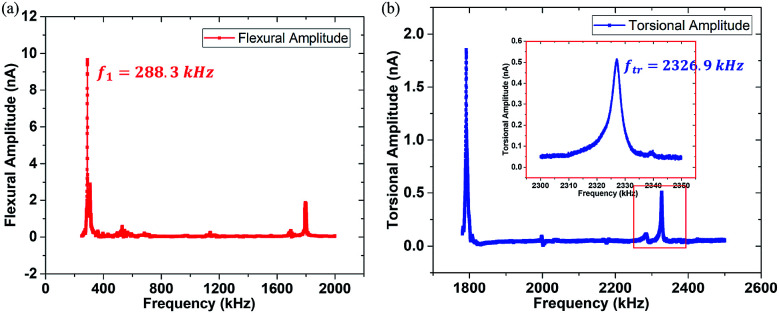
The frequency spectra (200–2500 kHz) of the cantilever PPP-NCH. (a) The flexural signal spectral response (red line) and the first order flexural vibration frequency *f*_1_ = 288.3 kHz. (b) The torsional signal spectral response (blue line) and the first order torsional vibration frequency *f*_tr_ = 2326.9 kHz. The inset is the details of the first order torsional resonance peak in the red box. The unit nA is an electric parameter in the AFM apparatus representing the amplitude response of the cantilever.

In order to obtain resonance peak frequencies from the spectra, it is necessary to extract the flexural and torsional response signals from the PSD device. The flexural amplitude sensitivity can be obtained in the flexural amplitude *versus* the tip-sample distance curve on a rigid Si surface at the end of the experiment, and lateral amplitude sensitivity can be calculated.^37,38^ They are 18.75 nm nA^−1^ and 2.14 nm nA^−1^, respectively. The resonant spectra of the two signals were displayed in Fig. 2, where the first order flexural vibration frequency *f*_1_ = 288.3 kHz and the first order torsional vibration frequency *f*_tr_ = 2326.9 kHz are shown, respectively. The cantilever used here is the PPP-NCH (*k*_1_ = 21.3 N m^−1^, nanosensors). The responding force constants are calibrated by Sader's method.^39^ The torsional force constant *k*_tr_ can be calculated to be 1387.8 N m^−1^.^26^ Typical parameters of the cantilever are listed in Table 1.

**Table 1:** Typical parameters of the PPP-NCH cantilever. The force constants are calibrated by Sader's method^39^

Manufacturer	Type	Bending			Torsion		
		*f*_1_ (kHz)	*k*_1_ (N m^−1^)	*Q*_1_	*f*_tr_ (kHz)	*k*_tr_ (N m^−1^)	*Q*_tr_
Nanosensors	PPP-NCH	288.3	21.3	286	2326.9	1387.8	515

## 3. Results and discussion

In bimodal DFM, the two excitation frequencies are exactly the two resonance peaks chosen to detect the sample. The rectangular cantilever beam can be approximately modeled by the Euler–Bernoulli partial differential equation to describe the dynamics of the cantilever-tip system. Then we obtain a system of two analytical expressions.^17,40^1
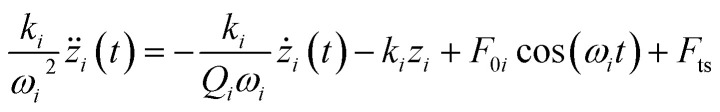
2
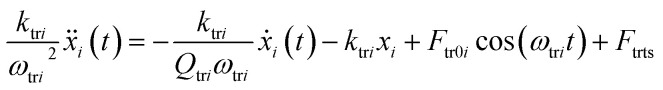
where *i* represents the *i*-th mode; *k*_*i*_, *ω*_*i*_ = 2π*f*_*i*_, and *Q*_*i*_ are the modal stiffness, resonance frequency, and quality factor of the *i*-th mode, respectively. *F*_0*i*_ = *k*_*i*_A_0*i*_/*Q*_*i*_ is the magnitude of the *i*-th mode external driving force. *A*_0*i*_ and *z*_*i*_ are the free amplitude and deflection of the cantilever for the *i*-th mode. *F*_ts_ and *F*_trts_ are the out-of-plane and in-plane tip-sample interaction, respectively. The subscript tr means the torsional vibration mode. Because the bimodal vibration mode contains the flexural and torsional motion, it can be separated into two decoupled forms to calculate the physical quantities, such as the dissipation and virial. The two directional vibrations can be solved to get the dynamics equations of the tip in the bimodal mode, as the following analytical formulas show:

3 *z*_*i*_(*t*) = *z*_0_ + *A*_*i*_ cos(*ω*_*i*_*t* − *φ*_*i*_)

4 *x*_tr_(*t*) = *A*_tr_ cos(*ω*_tr_*t* − *φ*_tr_)

where *z*_0_ is the mean deflection, *A*_*i*_ is the flexural amplitude and *φ*_*i*_ is the phase shift of the *i*-th mode. *x*_tr_ is the tip motion along the in-plane direction, *A*_tr_ is the torsional amplitude and *φ*_tr_ is the phase shift of the first order torsional mode, respectively.

**Fig. 3:**
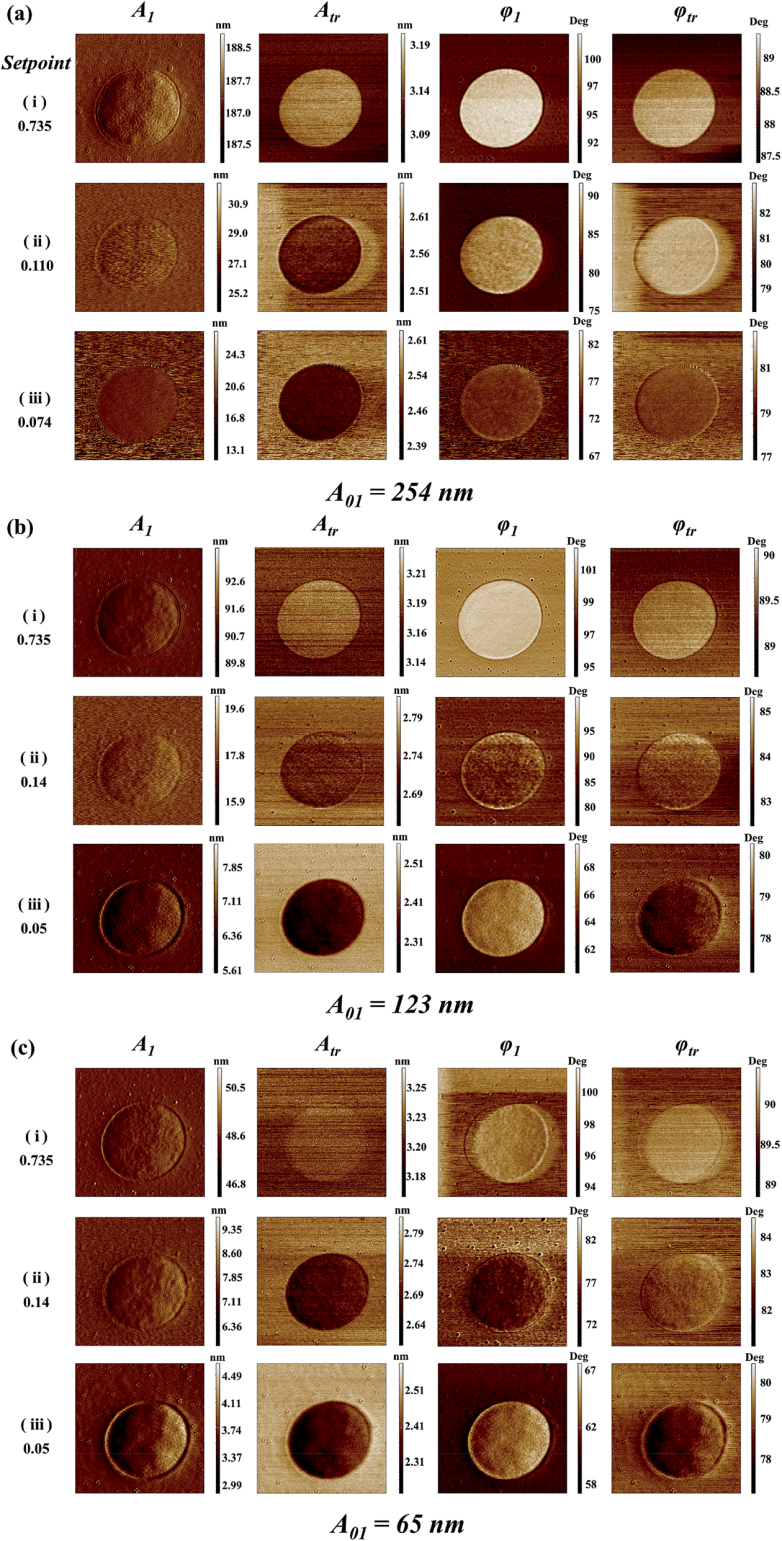
4.5 × 4.5 micrometer AFM signals of PS–LDPE imaged by an Nanosensors PPP-NCH cantilever in the bimodal AFM mode. Free amplitudes (a) *A*_01_ = 254 nm, (b) *A*_01_ = 123 nm and (c) *A*_01_ = 65 nm. The left value is the setpoint ratio. (i) Setpoint = 0.735, (ii) transitional setpoint, and (iii) setpoint = 0.074 in (a), 0.05 in (b) and (c) are the three chosen values of the setpoint ratio performed at the respective *A*_01_. The amplitude and phase images are in nm and degrees, respectively.

**Fig. 4:**
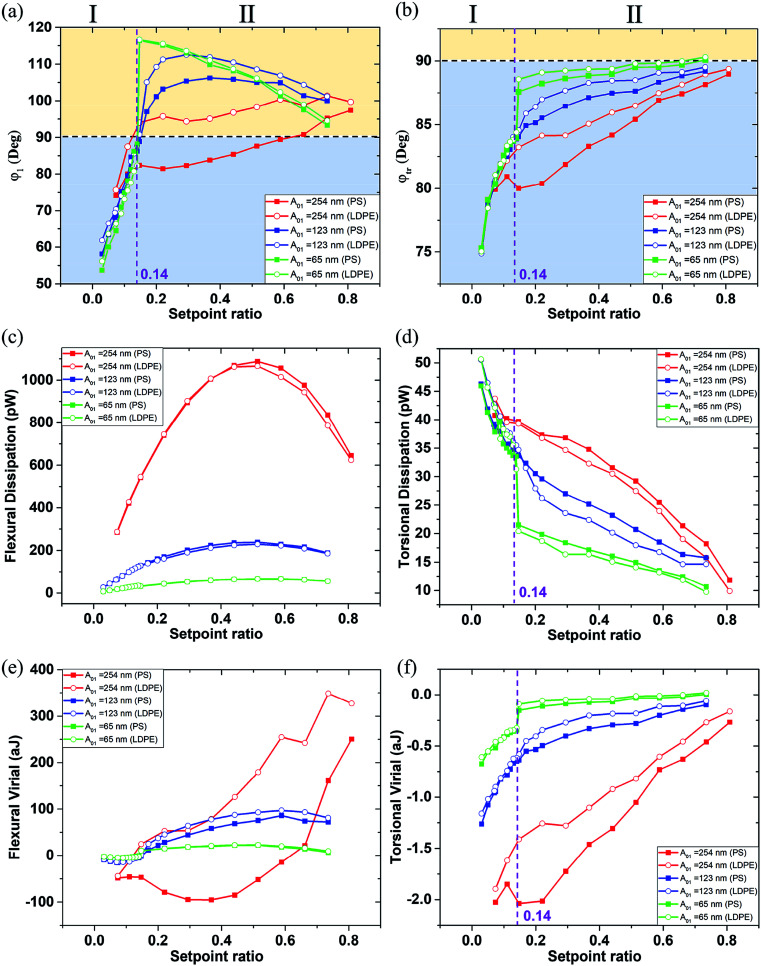
The phase, energy dissipation power and virial of the first order flexural and torsional signals under different free flexural *A*_01_ with the various setpoint ratio (0–1). The setpoint ratio axis is divided into two regions I and II in (a), (b), (d) and (f). (a) The first order flexural vibration phase *φ*_1_. (b) The first order torsional vibration phase *φ*_tr_. (c) Energy dissipation power of the first order flexural vibration mode. (d) Energy dissipation power of the first order torsional vibration mode. (e) Virial of the first order flexural vibration mode. (f) Virial of the first order torsional vibration mode. The first order free flexural vibration amplitude *A*_01_ is 254 nm (red line), 123 nm (blue line) and 65 nm (green line). The square dots and open circle dots represent the data on the PS and LDPE, respectively. The data points are the comprehensive reflection of the scanning image pixels by fitting the normal distribution. They are the data after error processing. The errors involved are less than the symbol size in all plots, but the data points are very close within the small setpoint ratio range, in which the experiment should be avoided because of the possible tip contamination.

Fig. 3 shows the bimodal excitations and scanning amplitude *A*_1_, *A*_tr_, phase *φ*_1_ and *φ*_tr_ images obtained in the bimodal experiments. The different free *A*_01_ (*A*_01_ = 254, 123 and 65 nm) are employed in the Fig. 3(a)–(c). The rows in Fig. 3(i)–(iii) represent the setpoint (0.735, transitional setpoint, and 0.074 in (a), 0.05 in (b) and (c), respectively). The transitional setpoint value of the setpoint are around 0.11–0.14 for different *A*_01_. For amplitude *A*_1_ images, no obvious color difference can be found except for the dark edges around the LDPE region, which may be caused by the topography variation. Therefore, the contrast of *A*_1_ is rather low because of the stable feedback mechanism. As the setpoint ratio decreases, the flexural phase *φ*_1_ difference of PS–LDPE reverses into three stages. In the first stage, the flexural phase *φ*_1_ image is bright in the LDPE region, and torsional phase *φ*_tr_ display a bright color at the large setpoint ratio. As setpoint ratio decreases to the second stage, the phase *φ*_1_ and *φ*_tr_ images between the PS and LDPE regions can only been distinguished under the large free amplitudes *A*_01_ = 254 nm in Fig. 3(a)-(ii), but not under the other two *A*_01_ in Fig. 3(b)-(ii) and (c)-(ii). In the third stage, the reversal of phase *φ*_tr_ occurs in Fig. 3(b)-(iii) and (c)-(iii), showing the torsional phase *φ*_tr_ is dark while the flexural phase *φ*_1_ is still bright in the LDPE region. In the meantime, the phase *φ*_1_ and phase *φ*_tr_ images are not sufficiently distinguishable in Fig. 3(a)-(iii). As for the torsional amplitude *A*_tr_, it experiences two stages, where the torsional amplitude *A*_tr_ image is bright in the LDPE region in the first stage but dark in the second stage. The high lateral force region leads to lower torsional amplitude (the darker region in Fig. 3). The sample mechanical properties, such as the surface adhesion energy, elastic modulus, stiffness, plasticity index or viscoelasticity, play an important role in the tip-sample interaction.^41^ In the first stage, the tip is vibrating far away from the surface, so the mechanical properties have little influence on torsional energy dissipation. However, in the second stage, the tip is close to the sample surface, resulting the lower torsional vibration on LDPE, mainly because the indentation on LDPE is larger than that on PS. The torsional dissipation power and virial difference between PS and LDPE components in Fig. 4(d) and (f) can also explain the reversal. The torsional dissipation power on LDPE are larger than that on PS in the setpoint ratio range less than 0.08, indicating that the torsional amplitude is more sensitive to mechanical properties of the specimens and the tip-sample distance.

### 3.1 Phase, dissipation and virial of the bimodal mode

In order to quantify the tip-sample interaction, the dissipation power and virial, which are derived from the phase and amplitude information, can be utilized to describe the conservative and dissipative interactions between the tip and the sample surface.^42,43^ They are convolutions of the tip sample interactions with position and velocity, respectively. The *i*-th mode average dissipation power *P*_dis_(*i*) and virial *V*_*i*_ per cycle can be calculated by the following analytical expressions:^44,45^5
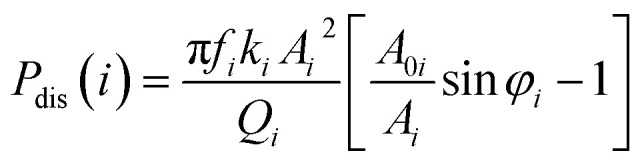
6
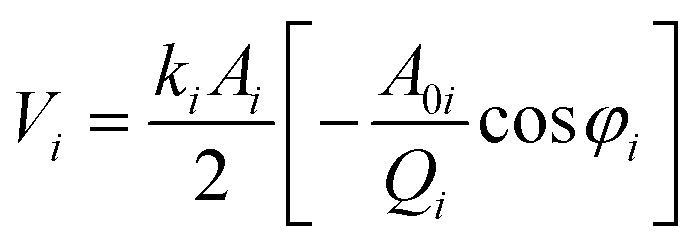
where *f*_*i*_ is the *i*-th mode free resonance frequency, *k*_*i*_ is the *i*-th mode force constant, *A*_0*i*_ is the *i*-th mode free amplitude, *A*_*i*_ is the *i*-th mode scanning amplitude, *Q*_*i*_ is the *i*-th mode quality factor, and *φ*_*i*_ is the *i*-th mode phase.

Bimodal experiments were conducted on the PPP-NCH cantilever by exciting the two vibration modes. In a series of experiments, the first order free flexural vibration amplitude *A*_01_ were kept as 254 nm, 123 nm, 65 nm, respectively. Because the maximum PSD lateral output was 1.6 nA, the first order torsional vibration was also driven maximum to 3.4 nm to better study the changes in the torsional energy dissipation *versus* the setpoint ratio of the flexural vibration mode in a wide range, where the different out-of-plane and in-plane interaction varied with the average distance between the tip and the sample surface.

Fig. 4 shows the phase, dissipation power and virial of the first order flexural and torsional signals under different free flexural *A*_01_ with the various setpoint ratio (0–1). In this article, setpoint is reported as a percentage of the free tapping amplitude. The region was divided by the 90° black dot line, where above is the attractive range and below is the repulsive interaction range.^41^ The setpoint axis was also divided into two sections I and II by the transitional boundary between the attractive and repulsive range. The phase *φ*_1_ curves are above the 90° line in the region II of Fig. 4(a) except for on the PS component with *A*_01_ = 254 nm, where the setpoint ratio is larger than 0.14. In the region smaller than 0.14, all the phases decrease rapidly below the 90° line, indicating that the interaction goes into the repulsive range because of the close distance and strong interaction between the tip and the sample surface. In Fig. 4(b), the phase *φ*_tr_ curves are all below the 90° line, indicating that the repulsive interaction dominates the in-plane interaction. A high free amplitude *A*_01_ can maximize the force at which the tip strikes the sample. The low setpoint, to an extent, is similar to the high free amplitude, both of which can enhance the tapping force. The viscous damping is closely related to the tip velocity. With increasing free amplitude or setpoint, the tip velocity and tapping force can be improved, so the effect of viscoelasticity becomes larger.^41,46^ When the free amplitude is very small, viscous damping is small and consequently the adhesion or the indentation between the tip and the sample should play a more important role in phase angle contrast. The repulsive interaction rapidly increases when the tip collides with the sample surface. The phase *φ*_tr_ decreases with the decrease of the setpoint ratio value. When the slope of the phase *φ*_tr_ increases within a low setpoint ratio range, it shows that the torsional phase decreases faster.

The dissipation power and virial of the bimodal vibration under different amplitude *A*_01_ in various setpoint ratio range are shown in Fig. 4. The flexural dissipation power seems a parabolic trend under a large *A*_01_ = 254 nm, and the maximum is around 0.5 setpoint ratio in Fig. 4(c). However, it remains steady under other two small *A*_01_. The flexural dissipation power of PS is larger than that of LDPE within most setpoint ratio range except for the setpoint range below 0.25, because the amplitude and phase are reversed in the close region I. The phase is an essential parameter to estimate the dissipation power in amplitude modulation AFM. The PS has a larger elastic module (*E*_PS_ = 2.0 GPa) than the low-density polyethylene (LDPE) (*E*_LDPE_ = 0.1 GPa). When the tip is touching the sample surface softly, it is harder for PS to produce the deformation. The tip retracts quickly on PS, so the phase shift is closer to 90 degree. The tip may press deep into LDPE, resulting a larger phase shift. From the phase curve in Fig. 4(a), the phase value on PS is closer to the 90 degree line, indicating the sin *φ*_*i*_ term of PS is larger than that of LDPE, so the flexural dissipative power on PS is larger than that on LDPE in most cases. Dissipation is very small at both ends of the setpoint ratio range because there the sample deformation is small and consequently the viscous force. The flexural virial on LDPE is smaller than zero at the beginning of the setpoint range, mainly because the tip is still working in the repulsive interaction range. After the flexural phase exceeds 90°, the flexural virial turns positive and the dominant tip sample forces are attractive. It has a continuous increase, and the flexural phase of LDPE is larger than that of PS. In Fig. 4(d), it is obvious that the torsional dissipation reaches the maximum in the region I, and rapidly drops till the transitional setpoint 0.14. In the region I, the torsional vibration near the sample surface results in large torsional dissipation. In Fig. 4(d) and (f), the abrupt increase of the torsional dissipation and virial of the green lines show that the tip seems to enter the strong repulsive interaction region when the setpoint ratio is lower than 0.14. The torsional vibration phase is lower than 90 degree and becomes more and more smaller nearly along the whole set-point range, indicating that the tip touches the sample from slightly to heavily. Although the flexural vibration phase ranges from 90 to 120 and then to 60 degree, when the setpoint ratio goes from 1 to 0. At the beginning of any given amplitude reduction, the flexural vibration as a whole exhibits the attractive interaction state. However, the contact phenomenon cannot be ruled out, because the contact force is extremely weak due to the short contact time and the small deformation. When the tip is far away, the contact and interaction are relatively weak, and the influence on the flexural vibration is small, but the torsional vibration is sensitive to the lateral micro-contact. At this time, the torsional dissipation of PS with large Young's modulus is more obvious. The interaction on PS is easier to become repulsive than that on LDPE, which is in consistent with the previous conclusion.^47^ The pull-in phenomenon caused by a strong attractive force, including van der Waals' force, the electrostatic force and the possible capillary force from a surface water film can occur near the transitional setpoint ratio value when the tip is close to the sample surface. From the phase images, the amplitude setpoint or called the transitional setpoint at which the mean force changes from overall attractive to overall repulsive is an unstable operation point. The equilibrium of attraction and repulsion is easily broken by a slight change in setpoint ratio, but once it is broken, especially for smaller free amplitudes, the torsional energy dissipation increases sharply. When beyond the transitional point 0.14, the flexural vibration is pulled out of the sample surface by the higher amplitude setpoint ratio, the torsional vibration works in a relatively weak repulsive interaction region, resulting a lower torsional dissipation and virial. In this case, the dynamic friction may be the main cause of the energy dissipation, and the shorter torsional vibration period near the sample surface can reduce dissipation and virial. The slope in the region I is much sharper than that in the region II, which is consistent with the steep torsional phase transition. However, both the absolute values of the dissipation power and the virial are larger under high free resonance amplitude *A*_01_. With increasing free amplitude, the effect of viscoelasticity becomes larger.

In ambient conditions, a thin film of water is likely to be absorbed on the sample surface. At close proximity of the tip and surface, a meniscus or liquid bridge may be formed between tip and sample.^48^ The meniscus implies an attractive (capillary) force. Nevertheless, the capillary force does not act at all distances, because it appears on the approach only at or shortly before contact. Retracting of the tip results in breakage of the capillary, thus elimination of the meniscus force at a significant distance from the surface. Therefore, the influence of water will be more important as the free amplitude becomes lower. At the nanometer scale, the capillary force between the tip and substrate is calculated as a function of their separation *h* was studied by molecular dynamics simulations.^49^ From the above description, as the setpoint ratio gets smaller and smaller until it reaches the transitional point. Due to the thin film of water, the average capillary force becomes larger, which is superimposed on the increasing van der Waals force, making the tip pull in the sample surface. In Fig. 4, the transitional point 0.14 represents three different scanning amplitudes, 35 nm, 17 nm and 9 nm, at the three free amplitude *A*_0_, respectively. They can be described by the continuum prediction due to the scanning amplitude larger than macroscopic height *h*_m_ (∼10 nm), so there is no doubt that the capillary force makes a contribution to the adhesion in addition to the van der Waals forces, which makes it easier for the tip to be attracted to the sample surface during close scan. The amplitude and phase values between PS and LDPE may be similar. However, the scanning amplitude 9 nm is so small that a quick phase reduction occurs. Therefore, the influence of water will be more important as the free amplitude becomes lower.

**Fig. 5:**
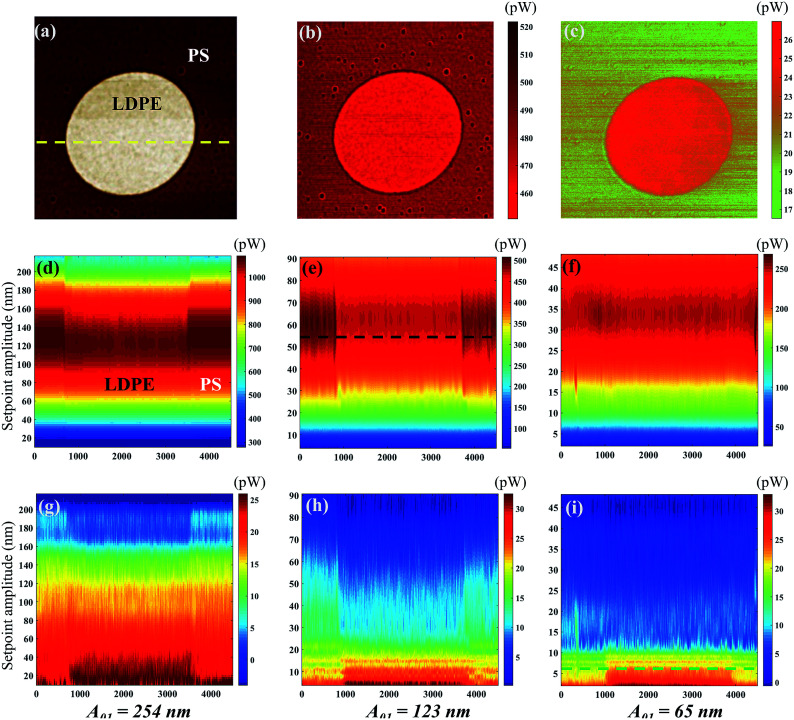
The sectional energy dissipation distribution. (a) Phase image of the PS/LDPE. (b) Cross sectional flexural dissipation distribution at the setpoint amplitude 54 nm (*A*_01_ = 123 nm) as shown by the black line in (e). (c) Cross sectional torsional dissipation distribution at the setpoint amplitude 6.5 nm (*A*_01_ = 65 nm) as shown by the green line in (i). The longitudinal sectional flexural dissipation distribution at (d) *A*_01_ = 254 nm (e) *A*_01_ = 123 nm and (f) *A*_01_ = 65 nm. The longitudinal sectional torsional dissipation distribution at (g) *A*_01_ = 254 nm (h) *A*_01_ = 123 nm and (i) *A*_01_ = 65 nm. The side length is 4.5 micrometer and the longitudinal sectional position of (d)–(i) is the yellow line in (a).

The distance dependence of the tip-sample energy dissipation has been recorded at different setpoint ratios to capture not only the spatial variation in the *X*–*Y* plane, but also as a function of the tip-sample distance to some extent. Fig. 5 shows the sectional energy dissipation distribution calculated from data in Fig. 4. Fig. 5(a) shows the phase image of PS (the dark brown area) and LDPE (the bright circular area). The flexural and torsional energy dissipation images of the yellow line in Fig. 5(a) are plotted in Fig. 5(d)–(i), from where we can see the tip-sample interaction is different on PS and LDPE, especially in the torsional dissipation. The material components are clearly identified. Compared to the data points in Fig. 4, the visualization is not a strict way to distinguish the two materials, but some differences can be intuitively seen. For example, the flexural dissipation power of the two materials is close to each other especially with in the low setpoint range at a small free amplitude 65 nm. It does show the absent difference in Fig. 5(f), however, the small differences in Fig. 5(d) and (e) are observable between PS and LDPE. The flexural dissipation power on PS is larger than that on LDPE. It is also found that the flexural dissipation of the first order at long distance (large setpoint amplitude) is generally greater than that at short distance, however, if the distance is too far, the energy dissipation will also be reduced. For the torsional dissipation distribution in Fig. 5(g)–(i), it is consistent with the trend in Fig. 4(d). The difference in torsional dissipation seems a bit better because the boundary between the PS and LDPE can be clearly seen in Fig. 5(g)–(i), especially within the region of setpoints lower than 0.14, where the torsional dissipation on LDPE is larger than that on PS. However, the opposite is true within the region of setpoints higher than 0.14, the torsional dissipation on LDPE is larger than that on PS. The cross sectional flexural dissipation in Fig. 5(b) is the detailed description of the black line in Fig. 5(e), where the flexural dissipation on PS is larger than that on LDPE. Fig. 5(c), the detailed description of the green line in Fig. 5(i), shows the near torsional dissipation distribution on LDPE is larger than that on PS.

### 3.2 Contrast of the amplitude and phase

To study quantitatively the contrast of amplitude and phase, the pixel values can be extracted and calculated the histogram of response from each AFM scanning image. Each pixel belongs to one of two classes, for example, PS or LDPE. It may appear as two distinct peaks which is a continuous probability distribution. The normalized histograms and the bimodal distribution function is fitted by^12^7
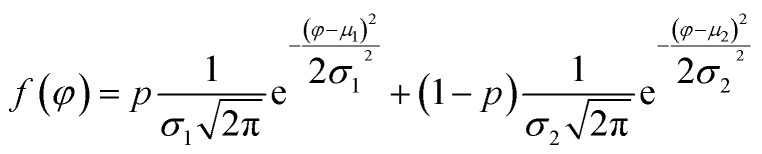
where *μ*_1_ and *μ*_2_ are the means of the two normal distributions of PS and LDPE, *σ*_1_ and *σ*_2_ are the standard deviations, *p* is the ratio of pixels in the first distribution and (1 − *p*) the ratio of pixels in the second. For bimodal distributions, the two main factors are the mean values and standard deviations to define the average and quantify the amount of variation or dispersion of a set of phase values (0 < *φ* < 180°). Ashman's *D* is used to quantify phase and amplitude image contrast, and *D* > 2 is a necessary condition for a clear separation of two mixed materials. *D* can be calculated by the following statistical formula^50^8
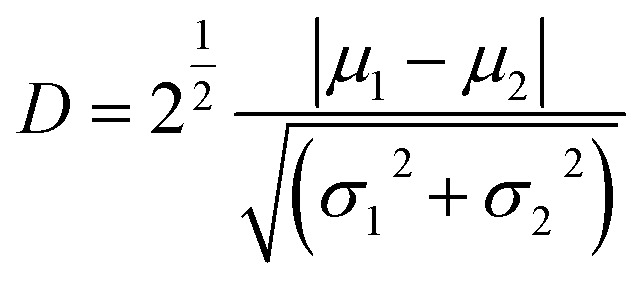


**Fig. 6:**
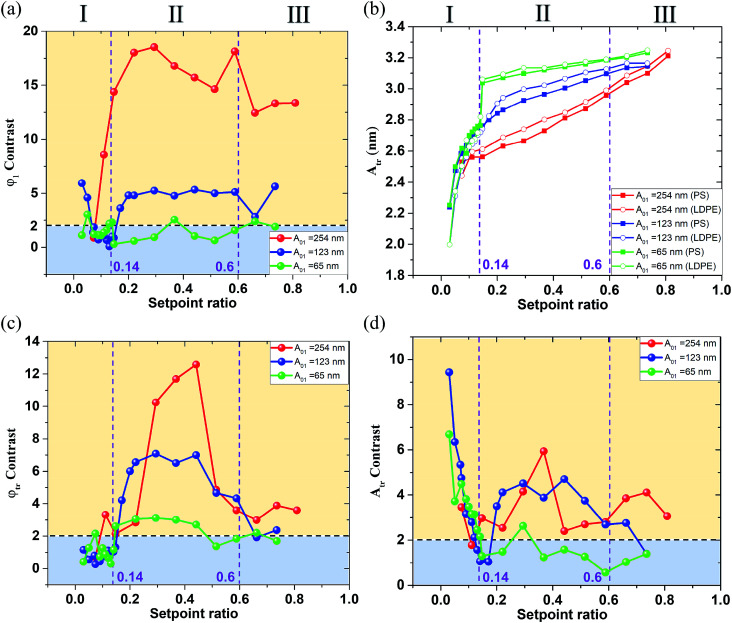
The amplitude and phase contrast of the first order flexural and torsional signals under different free flexural *A*_01_ in the various setpoint ratio range. The setpoint ratio axis is divided into three regions I, II and III in every image set. (a) The first order flexural vibration phase *φ*_1_ contrast. (b) The first order torsional vibration amplitude *A*_tr_. (c) The first order torsional vibration phase *φ*_tr_ contrast. (d) The first order torsional vibration amplitude *A*_tr_ contrast. The purple dotted lines are the boundaries between different contrast stage. The first order free flexural vibration amplitude *A*_01_ is 254 nm (red line), 123 nm (blue line) and 65 nm (green line).

From the experimental results above, the phase and amplitude contrast of the first order flexural and torsional signals under different free flexural *A*_01_ are displayed in Fig. 6. The phase *φ*_1_ contrast curves show a similar trend as the red and blue contrast curves are higher in the region II than in the region I and III of Fig. 6(a). The green contrast curve keeps a steady value below 2, meaning a failure criterion for a clear separation of the two mixed materials. The contrast seems more obvious at a large *A*_01_ and in the intermediate setpoint ratio region II of Fig. 6(a). The phase *φ*_1_ contrast at the *A*_01_ equal to 254 nm is 3–4 times larger than that at *A*_01_ equal to 123 nm. This can be explained by the degree to which the tip is able to penetrate and then deform the sample.

Consistent with the phase *φ*_1_ trend, the phase *φ*_tr_ abruptly reduces in the region I and III of Fig. 6(c). The three *φ*_tr_ contrasts are all higher in the region II than in the region I and III of Fig. 6(c), where the green *φ*_tr_ contrast curve keeps a steady value around 3, meaning a success criterion for a clear separation of the two mixed materials. The *φ*_tr_ contrast is also larger than the *φ*_1_ contrast when *A*_01_ is 123 nm and 65 nm in the region II, showing that low *A*_01_ can enhance the torsional phase contrast due to the longer interaction time.

Fig. 6(b) and (d) show the first order torsional amplitude *A*_tr_ and contrast under different free flexural *A*_01_ in the whole setpoint ratio range (0–1). The setpoint axis is also divided into three regions I, II and III in Fig. 6(b) and (d) by the contrast standard. The *A*_tr_ curves have slower slopes in the region II and III, and *A*_tr_ on LDPE is always larger than that on PS, indicating the PS material produces more interaction on the tip. In the region smaller than 0.14, all the torsional amplitudes decrease rapidly, mainly because the interaction goes into the strong repulsive interaction range. The torsional amplitude *A*_tr_ decreases with an increase in torsional dissipation. In the meantime, the contrast is contrary to the trend of the setpoint amplitude variation. It increases rapidly with the decrease of the setpoint ratio in the region I of the Fig. 6(d). The *A*_tr_ contrast curves show that the red and a small part of blue contrast curves in the region II are generally higher than those in the region I and III of Fig. 6(d). However, when the setpoint ratio is small enough, the blue and green contrast curves in the region I are higher than those in the region II of Fig. 6(d). The green contrast curve is lower than the criterion *D* = 2 in the region II of Fig. 6(d), meaning a failure criterion in the region II but successful for a clean separation of the two mixed materials in the region I of Fig. 6(d). However, the possible tip contamination is more likely to occur in the region I with low setpoint ratio value. When the indentation is deep, the tip tends to pick up sample impurities. For an exploratory experiment, in order to study the energy dissipation of different probe states, it is necessary to traverse the whole set value region, but the low setpoint ratio should be avoided in practical experiments, even though enhanced contrast of the torsional amplitude at low setpoints can be obtained. From the above analyses, in order to obtain a better contrast between both materials, the best approach is to work at intermediate setpoints in terms of both phase contrasts and also torsional amplitude contrast. A boundary point 0.14 where the torsional vibration motion goes into the strong repulsive interaction range together with the flexural vibration, shows that the pull-in phenomenon may occur. The rapid decrease of the *A*_tr_ indicates the repulsive interaction dominates the in-plane interaction. When the tip approaches close to the sample surface in a vibration form, the torsional motion of the tip may scrape the sample surface.

## 4. Conclusion

In the present study, a bimodal approach simultaneously exciting both the first order flexural and torsional eigenmodes of the cantilever has been developed to detect the contrast, the out-of-plane and in-plane dissipation between the tip and the polymer blend of PS and LDPE. The flexural and torsional signal spectra are obtained by sweeping frequency method and the resonance peaks are distinguished for specific excitations in the experiments. The scanning images of different stages are displayed to confirm that contrast reversals occur within a small setpoint ratio range. The amplitude and phase of the flexural and torsional response signals have been utilized to calculate the contrast, energy dissipation power and virial. Three free flexural amplitudes *A*_01_ have been exploited to explore the effects of amplitudes on the above physical quantities.

We have concluded that both the flexural and torsional signals of the cantilever can reflect the out-of-plane and in-plane interaction and the dissipation power between the tip and the sample surface. The free flexural amplitude *A*_01_ plays an important role in the magnitude of the contrast, the dissipation power and virial. Generally speaking, the specific values are enhanced in the large free flexural amplitudes *A*_01_ except for the torsional amplitude contrast. The setpoint ratio influences the trend of the physical quantity curves. The flexural vibration goes into repulsive interaction range within the setpoint range lower than the transitional point 0.14 and the torsional vibration is all in the repulsive interaction range. When in the setpoint ratio range less than the transitional point, the flexural and torsional phase together with the torsional amplitude sharply decrease, indicating that the vibration has entered the strong repulsive interaction range. The pull-in phenomenon by a strong attractive force can occur near the transitional setpoint ratio value due to the small force constant. Both phase contrasts and torsional amplitude contrast can be significantly enhanced in the intermediate setpoint ratio range, indicating that this kind of parameters is a proper choice to distinguish the compliant heterogeneous materials. The torsional energy dissipation power and virial show a rapid increase in the small setpoint ratio range, while the slope of the flexural energy dissipation power or virial does not change suddenly, the reason of which is that the torsional vibration is more sensitive to the close tip-sample distance. The in-plane dissipation is much lower than the out-of-plane dissipation, but the torsional amplitude contrast is higher when the tip vibrates near the sample surface. However, due to the possible tip contamination in some cases, the low setpoint ratio should be avoided in practical experiments, even though enhanced contrast of the torsional amplitude at low setpoints can be obtained. In three groups of experiments with different free amplitude *A*_0_, we set the amplitude value from high to low. However, every time the system runs at a low set value for a period of time, and then at a high setpoint, we can still get clear images of PS and LDPE, so the tip is not contaminated in our experiments. The average tip-sample distance can be controlled by the setpoint ratio to study the in-plane dissipation at different height levels, which is mainly due to the tip-sample dynamic friction and the sample properties. The experiment results are of great importance to optimize the operating parameters of image contrast and reveal the mechanism of friction dissipation from the perspective of in- and out-of-plane energy dissipation at different height levels, which adds valuable ideas for the future applications, such as the compliant materials detection, energy dissipation and the lateral micro-friction measurement and so on.

## Conflicts of interest

There are no conflicts to declare.

## Supplementary Material
